# Retraction Note: Purified umbilical cord derived mesenchymal stem cell treatment in a case of systemic lupus erythematosus

**DOI:** 10.1186/s40169-018-0207-4

**Published:** 2018-09-14

**Authors:** Christopher D. Phillips, Pornpatcharin Wongsaisri, Thein Htut, Terry Grossman

**Affiliations:** 1 Phillips Neurological Institute, LLC, P.O. Box 251, Las Marias, PR 00670 USA; 2 IntelliHealth Plus Clinic, 53 Soi Ruamsudee, Ploenchit Rd., Lumpini, Pathunam, Bangkok, 10330 Thailand; 3 Grossman Wellness Center, 2801 Youngfeld St. #117, Golden, CO 80401 USA; 4 Bumrungrad International Hospital, 33 Soi Sukhumvit 3, Klongtoey Nua, Wattana, Bangkok, 10110 Thailand

## Retraction Note: Clin Trans Med (2017) 6: 31 10.1186/s40169-017-0161-6

The Editor-in-Chief is retracting this article [1] because the authors have not obtained consent to publish from the patient whose details are described in this case report. The article has been removed to protect the patient’s privacy. Terry Grossman has stated that he was not an author on this article. Christopher Phillips, Pornpatcharin Wongsaisri and Thein Htut did not reply to correspondence about this retraction.
